# Supplementary material for: Comparative Study of Proximal Femur Bone Tumor Patients Undergoing Hemiarthroplasty versus Total Hip Arthroplasty: A Meta-Analysis
Source: J Clin Med. 2023 Feb 3;12(3):1209. doi: 10.3390/jcm12031209 (PMC9918064; doi:10.3390/jcm12031209)
Supplement: Supplementary file 1 [file jcm-12-01209-s001.zip › jcm-2168967-supplementary.pdf]

Supplementary Table S1: Newcastle– Ottawa Quality Assessment Scale for Cohort Studies

Authors	Represe nt-ativenes s of the exposed cohort	Selectio n of the non-expose d cohort	Ascertai n-ment of exposur e	Demonst -ration that outcome not present at start	Compara-bility of cohort (2 points)	Asses sment of Outco me	Follow-up length adequat e for outcome to occur	Adequacy of Follow-up of cohorts (accounted for non-index hospitals)	Total Score (9 points possi ble)
Zucchuni 2021 [21]	1	1	1	1	2	1	1	1	9
Mendez 2006 [2]	1	1	1	1	1	1	1	1	8
Jamshidi 2020 [22]	1	1	1	1	2	1	1	1	9
Ogilvie 2004 [13]	1	1	1	1	1	1	1	1	8
Gusho 2021 [23]	1	1	1	1	1	1	1	1	8
Zwart 1994 [14]	1	1	1	1	1	1	1	1	8

Supplementary Figure S1: Funnel plot for publication bias

(1) Dislocation

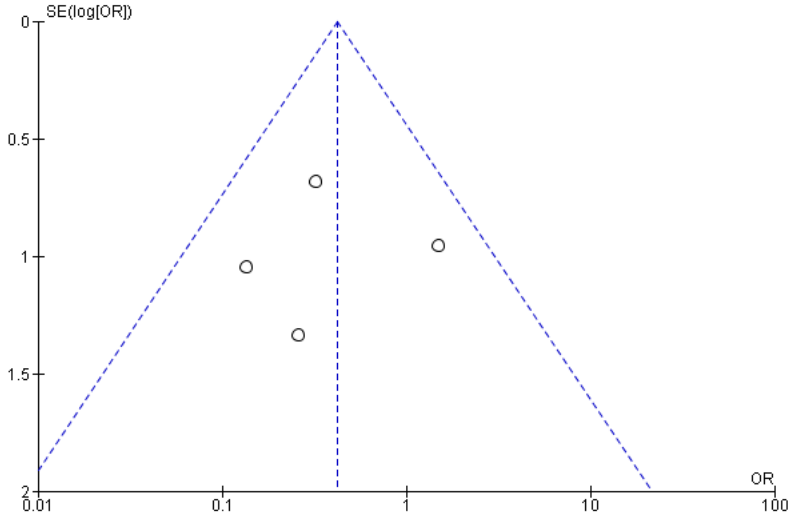

(2) Swing Phase

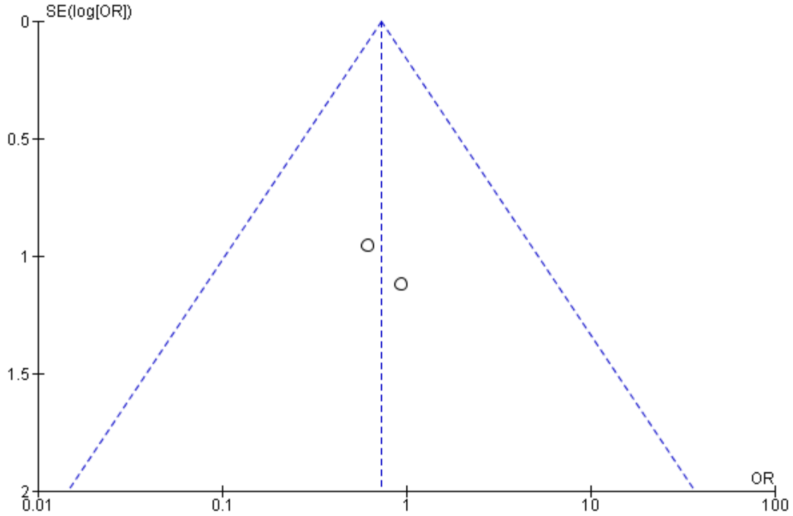

(3) Local recurrence

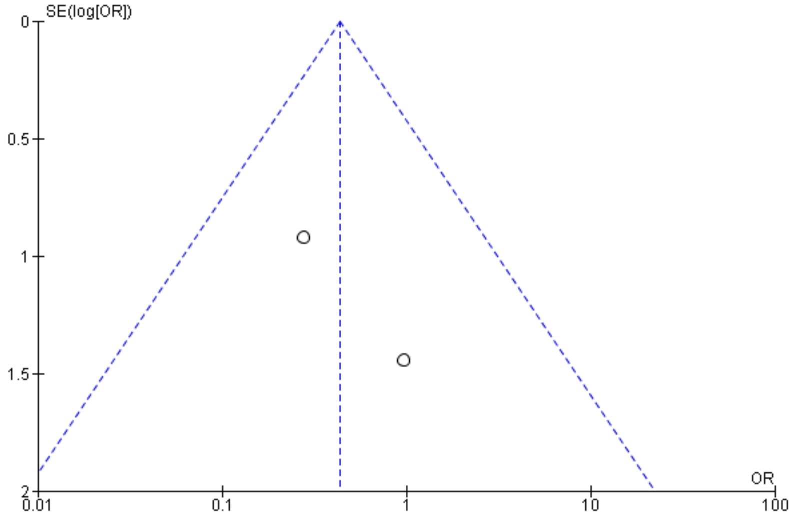

(4) MSTS

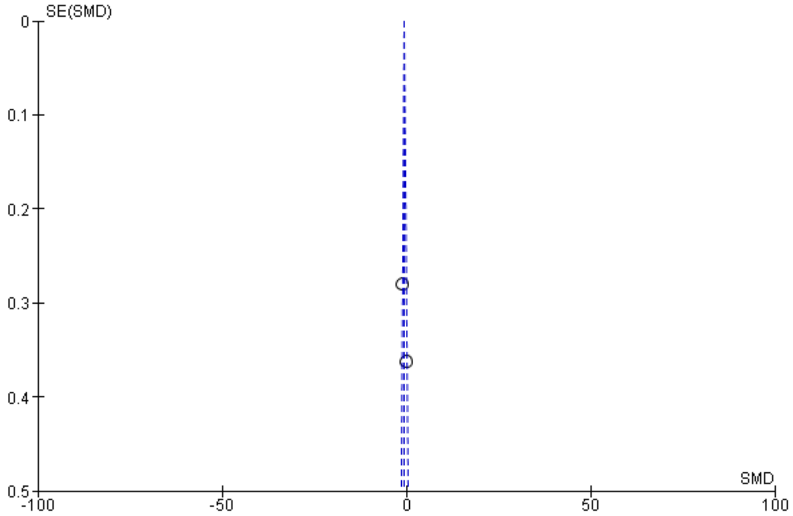
SE(SMD)
0
0.1
0.2
0.3
0.4
0.5
-100
-50
0
50
100
SMD

(5) HHS

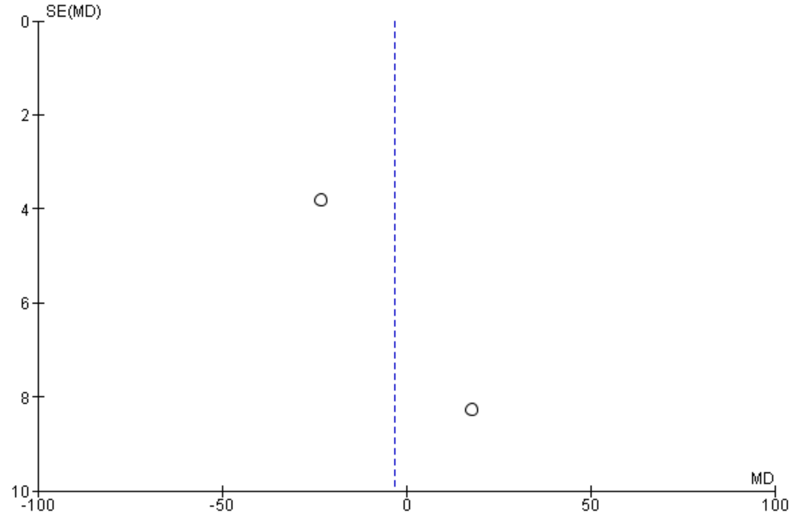
SE(MD)
0
2
4
6
8
10
-100
-50
0
50
100
MD

Supplementary Figure S2: Results of leave-one-out sensitivity analysis

Study or Subgroup	Experimental Events	Experimental Total	Control Events	Control Total	Weight	Odds Ratio M-H, Fixed, 95% CI
Gusho 2021	2	33	1	5	0.0%	0.26 [0.02, 3.53]
Jamshidi 2020	3	29	2	28	14.6%	1.50 [0.23, 9.73]
Menendez 2006	4	62	6	34	58.2%	0.32 [0.08, 1.23]
Zucchini 2021	2	90	2	14	27.2%	0.14 [0.02, 1.06]
**Total (95% CI)**		**181**		**76**	**100.0%**	**0.44 [0.17, 1.14]**
Total events	9		10			
Heterogeneity: Chi² = 3.12, df = 2 (P = 0.21); I² = 36%						
Test for overall effect: Z = 1.69 (P = 0.09)						

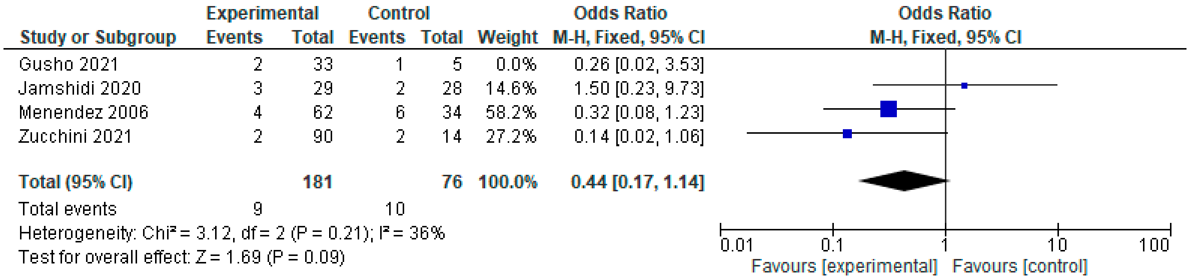

Supplementary Figure S2A: Forest plot for Dislocation after excluding study by Gusho et al. [23]

Study or Subgroup	Experimental Events	Experimental Total	Control Events	Control Total	Weight	Odds Ratio M-H, Fixed, 95% CI
Gusho 2021	2	33	1	5	13.3%	0.26 [0.02, 3.53]
Jamshidi 2020	3	29	2	28	0.0%	1.50 [0.23, 9.73]
Menendez 2006	4	62	6	34	59.1%	0.32 [0.08, 1.23]
Zucchini 2021	2	90	2	14	27.6%	0.14 [0.02, 1.06]
**Total (95% CI)**		**185**		**53**	**100.0%**	**0.26 [0.09, 0.75]**
Total events	8		9			
Heterogeneity: Chi² = 0.48, df = 2 (P = 0.79); I² = 0%						
Test for overall effect: Z = 2.50 (P = 0.01)						

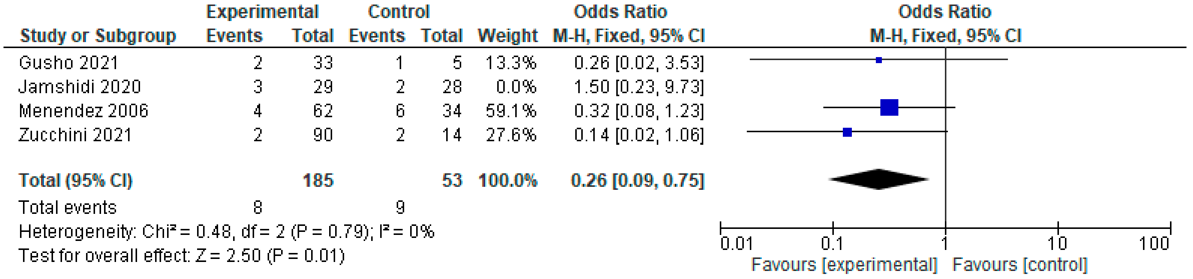

Supplementary Figure S2B: Forest plot for Dislocation after excluding study by Jamshidi et al. [22]

Study or Subgroup	Experimental Events	Experimental Total	Control Events	Control Total	Weight	Odds Ratio M-H, Fixed, 95% CI
Gusho 2021	2	33	1	5	23.9%	0.26 [0.02, 3.53]
Jamshidi 2020	3	29	2	28	26.7%	1.50 [0.23, 9.73]
Menendez 2006	4	62	6	34	0.0%	0.32 [0.08, 1.23]
Zucchini 2021	2	90	2	14	49.5%	0.14 [0.02, 1.06]
**Total (95% CI)**		**152**		**47**	**100.0%**	**0.53 [0.16, 1.77]**
Total events	7		5			
Heterogeneity: Chi² = 3.16, df = 2 (P = 0.21); I² = 37%						
Test for overall effect: Z = 1.04 (P = 0.30)						

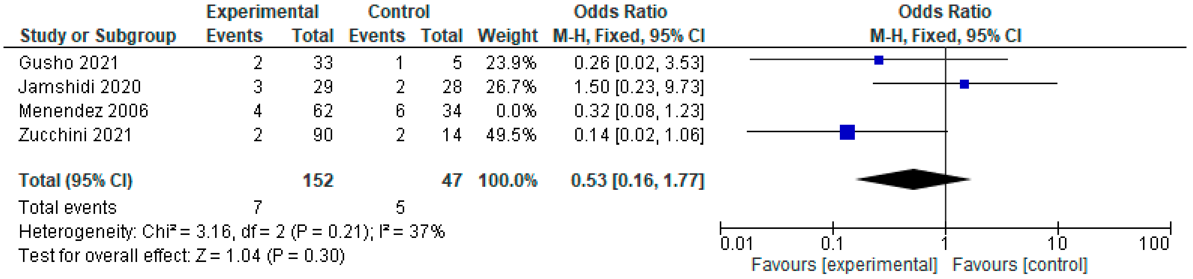

Supplementary Figure S2C: Forest plot for Dislocation after excluding study by Menendez et al. [2]

Study or Subgroup	Experimental Events	Experimental Total	Control Events	Control Total	Weight	Odds Ratio M-H, Fixed, 95% CI
Gusho 2021	2	33	1	5	15.2%	0.26 [0.02, 3.53]
Jamshidi 2020	3	29	2	28	17.0%	1.50 [0.23, 9.73]
Menendez 2006	4	62	6	34	67.7%	0.32 [0.08, 1.23]
Zucchini 2021	2	90	2	14	0.0%	0.14 [0.02, 1.06]
**Total (95% CI)**		**124**		**67**	**100.0%**	**0.51 [0.19, 1.37]**
Total events	9		9			
Heterogeneity: Chi² = 1.99, df = 2 (P = 0.37); I² = 0%						
Test for overall effect: Z = 1.33 (P = 0.18)						

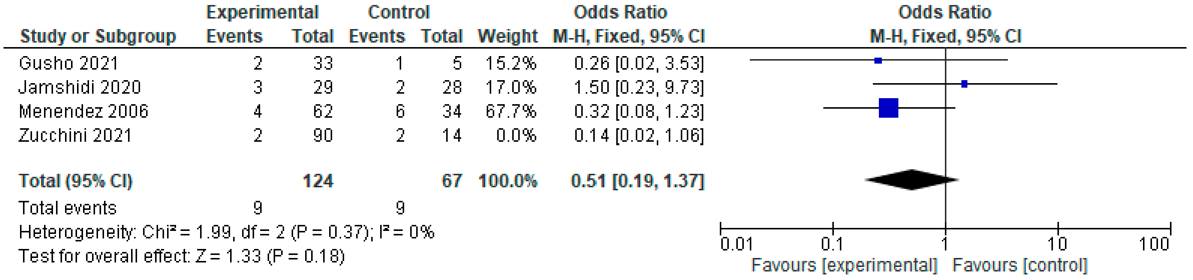

Supplementary Figure S2D: Forest plot for Dislocation after excluding study by Zucchini et al. [21]

References:

2 Menendez, L.R.; Ahlmann, E.R.; Kermani, C.; Gotha, H. Endoprosthetic Reconstruction for Neoplasms of the Proximal Femur. *Clin. Orthop. Relat. Res.* **2006**, *450*, 46–51. https://doi.org/10.1097/01.blo.0000229332.91158.05.

13 Ogilvie, C.M.; Wunder, J.S.; Ferguson, P.C.; Griffin, A.; Bell, R.S. Functional Outcome of Endoprosthetic Proximal Femoral Replacement. *Clin. Orthop. Relat. Res.* **2004**, *426*, 44–48. https://doi.org/10.1097/01.blo.0000136840.67864.78.

14 Hendrik, J.J.; Zwart', A.H.M.T. Johan W Schimme12 and Jim R van Horn2. Kotz modular femur and tibia replacement 28 tumor cases followed for 3 (1-8) years. *Acta Orthop. Scad.* **1994**, *65*, 31 5–31 8.

21 Zucchini, R.; Sambri, A.; Fiore, M.; Giannini, C.; Donati, D.M.; De Paolis, M. Megaprosthesis Reconstruction of the Proximal Femur following Bone Tumour Resection: When Do We Need the Cup? *Hip Pelvis* **2021**, *33*, 147–153. https://doi.org/10.5371/hp.2021.33.3.147.

22 Jamshidi, K.; Mirkazemi, M.; Gharedaghi, M.; Izanloo, A.; Mohammadpour, M.; Pisoudeh, K.; Bagherifard, A.; Mirzaei, A. Bipolar hemiarthroplasty versus total hip arthroplasty in allograft-prosthesis composite reconstruction of the proximal femur following primary bone tumour resection. *Bone Jt. J.* **2020**, *102-b*, 524–529. https://doi.org/10.1302/0301-620x.102b4.bjj-2019-0925.r2.

23 Gusho, C.A.; Clayton, B.; Mehta, N.; Colman, M.W.; Gitelis, S.; Blank, A.T. Survival and outcomes of modular endoprosthetic reconstruction of the proximal femur for primary and non-primary bone tumors: Single institutional results. *J. Orthop.* **2021**, *25*, 145–150. https://doi.org/10.1016/j.jor.2021.05.008.
